# Cultured Meat Prospects for a Billion!

**DOI:** 10.3390/foods10122922

**Published:** 2021-11-25

**Authors:** Sishir K Kamalapuram, Harish Handral, Deepak Choudhury

**Affiliations:** 1 Sanjeevani BioServices Pvt Ltd., Hyderabad 500084, India; sishir.bioprinting@gmail.com; 2 Biomanufacturing Technology, Bioprocessing Technology Institute (BTI), Agency for Science, Technology, and Research (A*STAR), 20 Biopolis Way, Singapore 138668, Singapore; harish_handral@bti.a-star.edu.sg

**Keywords:** cultured meat, cultivated meat, cellular agriculture, alternative protein, meat substitute, plant-based meat, food production, sustainability, consumer behaviour, India

## Abstract

The dietary protein requirements of almost 9.8 billion people need to be fulfilled in a healthy and sustainable manner by 2050. Meat consumption contributes to 35% of the total protein requirement of the Indian population. Meat intake needs to be sustainable and economical without causing food security and production issues. Consumption of meat in India is projected to rise with an increase in consumer incomes. Hence, novel alternative proteins, including cultured meat (CM) and plant-based meat (PBM), are being developed to satisfy the demand for meat-derived proteins in the diet. This involves the creation of novel PBM/CM products with a similar taste and texture as conventional animal meat with tailor-made nutritional attributes. In this article, we provide critical insights into the technical and business aspects of relevance to production and sustainability encountered by the Indian CM industry at a series of stages that can be termed the CM value chain comprising upstream and downstream processes. We shed light on the need for regulatory authorities and a framework. Consumer concerns towards CM products can be alleviated through effective scientific communication strategies, including prior familiarity, narrative building and transparency, and labelling aspects of CM products.

## 1. Introduction

**Figure 1:**
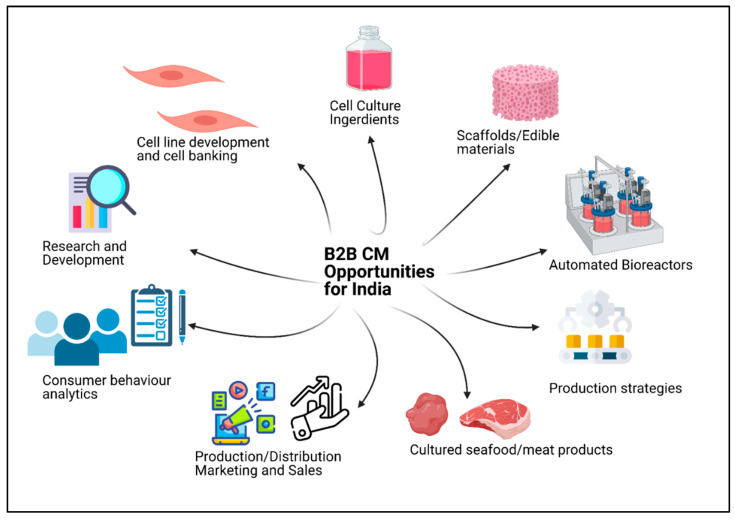
B2B CM opportunities for India (created with Biorender.com).

**Figure 2:**
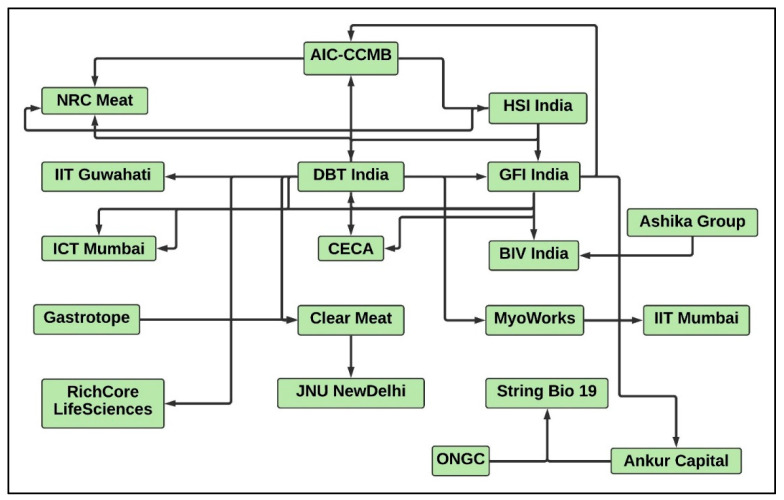
Current Indian CM ecosystem map (created with Lucidchart.com): arrows represent publicly reported collaborations between organisations/companies. DBT India, Department of Biotechnology, India; AIC-CCMB, Atal Incubation Centre—Centre for Cellular and Molecular Biology; ICT Mumbai, Institute of Chemical Technology (ICT) Mumbai; CECA, Centre of Excellence in Cellular Agriculture; NRC Meat, National Meat Research Centre; GFI India, Good Food Institute India; HSI India, Humane Society International (HSI) India; BIV India, Big Idea Ventures India; IIT Guwahati, Indian Institute of Technology Guwahati; ONGC, Oil and Natural Gas Corporation.

There is a dire need to fulfil the dietary protein demands of the projected population of 9.8 billion people in a sustainable and healthy manner by 2050 [1]. The fourth industrial revolution, which includes the digitalisation and automation of industrial manufacturing technologies, can foster the development of novel dietary proteins in order to address the food sustainability and security issues of the increasing population [1]. Food security issues are prevalent among India’s growing population and can be attributed to limited cultivation land, unsustainable food production, climate change, and governmental policies for the public distribution and marketing of food products [2]. Meat consumption in India contributes to 35% of the total protein requirement [3] with a production estimate of 7361 thousand tonnes in 2021 by the Food and Agriculture Organisation of the United States [4]. The consumption of meat in India is projected to rise with an increase in consumer incomes [5]. Novel alternative proteins, including cultured meat (CM) and plant-based meat (PBM), are being developed to satisfy the demand for meat-derived proteins in the diet [6]. CM is derived from in vitro cell growth of relevant animal species and involves the development of products similar to conventional meat [7]. PBM is the fabrication of meat-like products by utilising plant-derived components [6]. The cultured meat sector is projected to minimise the gap between the existing demand and supply of meat-derived proteins among the Indian population. Conventional animal meat products can be substituted by novel PBM or CM products with a similar taste and texture appeal, a tailor-made nutritional profile, low saturated fats, and a high fibre quotient with a negligible risk of contamination and a minor amount of antibiotics [1,8]. A rise in the average income of consumers and the gross domestic product (GDP) of India may enable its population to purchase affordable, hygienic, and healthy CM products by addressing the issues of the prevailing pandemic situation, health concerns, food adulteration, food sanitisation, violation of ISO standards and hygiene, and the inadequate traditional meat supply chains [9,10]. One of the long-term business goals for the CM industry in the Indian and global market is the production of economically affordable and sustainable CM products with a reduction in the cost of raw ingredients by the implementation of a cost-effective scale-up strategy [11,12,13]. Existing large companies and startups working in PBM alternatives and the CM sector are mostly self-integrated and create in-house solutions for each technical challenge without significant collaborations with their counterparts [14,15,16]. The CM industry is anticipated to become more diverse in the near future. The key to commercialisation and making CM an economically sustainable option is to encourage business-to-business (B2B) collaborations and industry–academic research partnerships in terms of speciality or product focus as the industry begins to become more diverse (Figure 1) [8]. On par with technological advancements, significant progress on the regulatory front is also essential in order to create a fair and transparent regulatory framework for the propagation of the CM market and the sale of designated CM products in India and abroad [17,18]. The Indian government has recognised CM as a sustainable and scalable approach for meat production in the alternative protein category [19]. Such exciting and ground-breaking advancements are a pressing priority, and critical driving factors including government investments, investor interest, and supportive consumer perspectives are to be considered [20]. In addition to startups, established companies in the life sciences, agri-food, and therapeutics sectors are in the race to acquire India’s CM market [21]. The development of the CM industry includes a series of stages (Figure 1) that can be termed the ‘CM value chain’, comprising: (1) upstream production stages ((a) research and development, (b) CM cell line development and cell banking, and (c) industrial-scale manufacturing strategies for the production of cell culture ingredients, edible scaffold materials, automated bioreactors, cultured meat, and sea food products); and (2) downstream production stages ((d) manufacturing and production facilities, (e) sales and distribution, (f) supply chain management, and (g) regulatory and business). The emerging CM industry in India has already started to show signs of maturity at a nascent level, as evidenced by several in-house pilot-scale proof of concept models and B2B collaborations for industrial-scale development at various entry points as represented in the Indian CM value chain (Table 1) and the Indian CM ecosystem map (Figure 2) [20,22]. The CM market has been envisaged to experience the most accelerated growth, offering significant options in the domains of investments, business, and research [23]. It is important to foster a robust market environment in the Indian and global startup ecosystem to boost the competitive technological landscape in CM and maximise niche markets by radically reforming the way meat is produced and processed [23]. The current startup ecosystem is largely integrated and determined to support in-house meat processing strategies at all possible stages of CM development without sufficient B2B partnerships [14,16]. However, business opportunities exist at all stages of the CM value chain, including: (a) upstream production stages (ingredients needed for CM fabrication and CM product development and manufacturing); and (b) downstream production stages (marketing, supply chain management, and delivery and food services) [14,16]. In this review, we delineate sensitive intervention points in developing the CM value chain ecosystem for India and highlight notable technical and business aspects of relevance to CM production, CM product focus, the regulatory framework, consumer behaviour, concerns over CM, and the future outlook for the Indian CM market.

## 2. Need for Cultured Meat in India

India is anticipated to be one of the world’s most populated countries and have one of the world’s largest economies, with the expected surge in meat consumption over the next 30 years having a significant impact on the global food system and environment [6]. An imbalance has been noted in: (a) the demand–supply aspect of meat consumption patterns; (b) the limited availability of livestock husbandry; and (c) the depletion of grasslands and forest cover due to the overgrazing of animals, which poses environmental concerns [24]. Meat consumption offers nutritional benefits along with potential health risks associated with unhygienic meat processing and supply chain management practices [9]. Currently, the price of meat in India ranges between 150 and 2000 Indian rupees (INR) per kilogram (kg), which is significantly higher than the price of 1 kg of vegetarian food. Increased meat consumption tends to create global opportunities for alternative protein (cultured meat, plant-based meat) markets, allowing the food industry to re-strategise sustainable future foods in realms of existing food security challenges [24,25]. Variants of PBM products, including mock meats, pizza, minced meat, and nuggets, have garnered substantial interest from Indian consumers, leading to the promotion of dynamic entrepreneurship platforms in the Indian PBM sector [26,27]. This is evidenced by the notable increase in novel plant-based meat startups, including GoodDot, Imagine meat, and Wakao foods, in the Indian market [27]. These exciting developments will undoubtedly instil curiosity in stakeholders and consumers about the CM product variants to be developed with respect to the diversity and uniqueness of the Indian food palate. The evolving Indian CM industry needs to focus on: (a) the production of affordable and sustainable CM products; (b) CM products with tailor-made nutritional aspects; and (c) a reduction in the environmental effects of the growing Indian CM industry [9]. There is a dire need for the nascent Indian CM industry to fulfil the aforementioned requirements in order to surpass the current limitations of the conventional meat industry. Otherwise, cultured meat will be classified as a premium and costly food option for elite or rich community members. 

## 3. Business Aspects of the CM Industry

The potential for the success of the CM market is primarily based on scientific advances from both industry and academia. A possible target for the Indian CM industry is a reduction in both upstream and downstream production costs by the creation of robust and flexible bioprocesses [23]. India’s CM industry is expected to diversify in terms of company specialisation and business creation opportunities and emphasise research and development (Figure 1) [7,28]. Large companies and startups in the CM sector are primarily focused on technical challenges, including cell line optimisation, the design of cost-effective procedures such as procedures for the production of media variants, scaffolding methodologies, and the design of scalable bioprocesses [8]. It would certainly be advantageous for leading life sciences and protein manufacturing firms to create specialised divisions and cultivate B2B partnerships along with CM value chain entry points [29,30]. Multinational life-sciences-related and biomanufacturing companies could set up their accelerator or incubator programs for designing novel CM ingredients in India. One such example is the Merck group’s creation of a “Clean Meat Innovation Field” at the Merck Innovation Hub in Silicon Valley, CA, USA [31,32]. Indian research organisations could accelerate the transfer of technology in the CM domain by promoting open-access and shared resources, including manufacturing protocols, cell line banking, scaffolding, and tissue engineering concepts [23]. The public sector and companies in India should facilitate the development of consortiums similar to the non-profit Cultivated Meat Modeling Consortium (CMMC) in Seattle, WA, USA in order to increase industry-academia cooperation, accelerate technological developments related to CM, establish new in silico models for examining cost reduction factors, and estimate timelines for resolving CM-specific technicalities [33].

Startups and established companies in India are indeed uniquely placed to capitalise upon this massive CM market opportunity. Startups are best positioned to spin off new products and technologies due to their agility and adaptability [34,35]. Working with established firms can add value as it makes use of their expertise, resources, and skilled professionals in product development, scale-up, production infrastructure, marketing strategies, distribution, and sales paradigms for the startup sector [20,35]. Increasingly, startups and established companies are engaging in mutual collaboration and skill strengthening, as demonstrated in the Indian CM ecosystem map (Figure 2). There is a quintessential need to set up accelerator units that help small and new brands in order to boost long-term innovation strategies [30]. For example, alternative protein firms in PBM or fermentation sectors might be best positioned to standardise specific parameters and develop desirable CM-based ingredients. Regardless of the species or strain of origin of designated CM products, raw material, accessorial support service, and technology development will be commonplace [36]. Proactive institutions, organisations, and startups contributing to business, the regulatory framework and technology enhancement in the Indian CM industry are listed in Table 2.

## 4. Technical Aspects of the CM Industry

### 4.1. Upstream Processes

#### 4.1.1. Custom Optimisation, Formulation, and Production of Media

The in vitro culture of animal cell lines, such as muscle cells, requires an appropriate medium and essential nutrients as growth substrates, leading to the formation of meat tissue [47]. There is a basic need for affordable and suitable cell culture media, which would significantly drive large-scale CM production [17]. Currently, most Indian firms conducting research and development in the CM domain utilise biomedical-grade media. Nevertheless, this will never be a feasible option for industrial-scale manufacturing as it incurs high costs for the food industry [48]. Established firms currently manufacturing media formulations for cell culture research and biomedical companies could come to develop serum-free media formulations for the CM industry [49]. RichCore Lifesciences Pvt Ltd. is working on the development of serum-free media for the in vitro growth of cultured meat cells [21].

#### 4.1.2. Cell Line Development and Cell Banking Strategies

Cell line development and cell banking facilities act as a repository for cultured meat cell lines and ensure long-term propagation of the CM industry in India and abroad [47]. Cell culture and cell banking companies in India can develop custom-designed species-specific (e.g., chicken, goat, sheep, fish, and shrimp) cell lines with a plethora of attributes, including robust cell lines for high-throughput screening, growth-factor-independent cell lines, the predisposition of cells to differentiate towards a specific cell lineage, and suspension-culture-tolerant cell lines, and, most importantly, adapt them for large-scale cell manufacturing [50]. Further, firms may need to design tailored standard operating protocols relevant to market trends and comply with regulations on cell line establishment, carbon footprint-free immortalisation techniques, and maintenance and cell banking aspects [51]. The Atal Incubation Centre—Centre for Cellular and Molecular Biology (AIC-CCMB) and the National Research Council for Meat (NRC Meat) have been working on the isolation and development of CM cell lines from goat species [19,23].

#### 4.1.3. Customised Bioreactors for CM

Bioreactors are containers that ensure optimal conditions for the growth of CM cell lines in either continuous or batch-wise operation [47]. Perfusion bioreactors ensure the continuous culturing of cells and are most commonly employed in the cell culture industry [47]. The flow of media and nutrients through the growing meat tissue placed on a growth-supporting biomaterial (scaffold) in these bioreactors offers high-quality end products [47]. Hence, designing large-scale perfusion bioreactors with media recycling technology to ensure minimal inputs and wastage and real-time quality control systems for balancing pH, osmotic homeostasis, temperature, and pressure is an immediate need for the commercial production of CM in India [47,49]. However, the fabrication of customised bioreactors for CM might require close collaboration between scaffold manufacturers and bioreactor firms in order to obtain the required expertise when designing innovative bioreactor models [49].

#### 4.1.4. Scaffolds for CM Applications

Scaffolds are biomaterials that mimic the physiological conditions of the extracellular matrix of a tissue environment in an in vitro setting [47]. Scaffolds provide structural support for CM cell attachment and growth, which can lead to the formation of meat tissue in vitro [47]. There is a need for the production of commercial-scale, low-cost, porous, high-quality, and edible-food-grade scaffolds (e.g., soybean, starch) for the growth of CM cell lines by employing technologies such as engineered hydrogels, 3D printing [52], spun fibre platforms, nanofiber systems, and extrusion technologies [17,53]. Fine-tuning technical parameters, including (a) growth medium penetration; (b) vascularisation; (c) stiffness; (d) cell adherence; and (e) the controlled release of growth factors can efficiently promote cell differentiation along the predefined axes of the scaffold. These modifications can help to achieve the desired tissue appearance, for example, the marbling finish observed on a slice of meat steak or the flakiness noticeable in a fish fillet [49,54]. The scaffold market in India would obtain significant B2B opportunities in the generation of scaffolds customised to the CM end product and outsourced to core CM manufacturers, resulting in a timeline reduction for CM product development. Myoworks Pvt Ltd., which was incubated at the Indian Institute of Technology, Mumbai, is working on the development of edible and low-cost scaffolds [44].

#### 4.1.5. Plant-Based Proteins with Better Functionality for CM Applications

Protein extracts derived from plants, including soybean, cereals, millets, and grains, are employed in the fabrication of edible scaffolds, textured proteins, and protein additives, which are currently utilised as ingredients in the fabrication of CM products and blended meats [9]. Suitable plant-based proteins can be developed as alternative protein ingredients to be utilised in CM product development with the following crop production attributes [9]. First, an increase in the productivity quotient, robustness, and resistance to biotic/abiotic stress to decrease agricultural expenses. Second, the development of crop variants tolerant to a wide range of climatic conditions, including temperature, drought, and salinity, for growing opportunities at various geographical locations in India and to ensure a seamless supply chain. Third, an enhancement of the protein content to ensure an improvement in functionality and the nutritional profile with increased bioavailability of macronutrients and micronutrients. Fourth, reconsideration of the flavour profile by the removal of off-flavours and the introduction of desirable Indian flavours in proteins. Fifth, plant proteins tend to have beany and grassy off-flavours due to the presence of compounds such as lipoxygenases, isoflavones, and saponins; so, the removal of off-flavours ensures that the quality and taste profile of CM ingredients are suitable. Finally, this sector can promote B2B opportunities in the CM ingredient sector by the utilisation of genetic engineering and/or high-throughput breeding approaches and the development of additives for blocking/neutralising off-flavours, biological techniques (e.g., microbial fermentation), chemical methods (e.g., incubation with an enzyme such as aldehyde dehydrogenase (ALDH)), physical methods (e.g., heating and cooling), and bioprocessing techniques (e.g., salting-out and β-Cyclodextrin-mediated methods) [9].

#### 4.1.6. Protein Variants as CM Ingredients

Textured protein variants are pre-extruded textured biomaterials specifically developed for the CM industry and can be utilised in the fabrication of scaffolds and protein additives with custom-designed flavours tailored to a CM product [53]. There is a need to broaden the range of protein varieties and textured plant-based proteins to be utilised as ingredients in CM product development [53]. Instead of utilising powdered protein concentrates and creating a fibrous structure through a protein extruder, CM manufacturers can consider pre-extruded textured proteins to be a more viable and economical option for CM ingredients [55]. For example, pre-extruded soybean scaffolds, which are widely utilised for the production of PBM, proved to be economical and technically feasible as a scaffold material [56]. Furthermore, specific variants of textured proteins can be fabricated by modifying them to resemble the meaty taste of conventional meat. The development of novel textured proteins could pave the way to extensive B2B opportunities in the CM ingredient sector [9]. The fabrication of novel textured proteins of different shapes, particle sizes, and custom-designed flavours can be achieved by the utilisation of the rich diversity of India’s protein resources [53].

#### 4.1.7. Characterisation of CM Ingredients

CM manufacturers might encounter inconsistencies in the quality of ingredients, such as scaffolds, protein concentrates, and CM additives, when they obtain from B2B suppliers. Hence, appropriate diagnostic kits, tools, and metrics have to be available for the characterisation of ingredients, including the particle size, colour, percentage of fat content, protein concentration, moisture content, and solubility quotient in varying pH ranges [53]. Furthermore, IP aspects of these characterisation methodologies can be licensed to CM ingredient and product manufacturers to enable the inclusion of these standard operating procedures (SOPs) into their quality control (QC) protocols for more qualitative measures [14].

#### 4.1.8. Supply of Fermented Ingredients for CM Applications

B2B opportunities can be promoted for the development of cost-effective fermentation-based CM ingredients, such as biomolecules, proteins, non-animal-derived fats, enzymes, and growth factors [57]. Fermentation-derived ingredients can be designed to improve the functional profile through novel additives and enhance the sensory appeal by optimising Indian flavours. Novel fat-encapsulated fermentation-derived compounds can be formulated to enrich the taste and texture appeal of CM and recreate the mouthfeel associated with the eating of meat [57].

#### 4.1.9. Characterisation and Sensor Technologies

There is a constant need to develop suitable and reliable sensors for the consistent and accurate real-time monitoring of the CM manufacturing process in terms of: (1) physical inputs (raw materials, essential ingredients, and water); (2) environmental conditions (temperature, pressure, and stress); and (3) the quality of the CM end product (size, morphology, colour, protein and fat concentration, fibrousness, consistency, taste, and nutrient profile) [47]. Hence, sensors offer advantages in evaluating the quality and quantity of CM products, thereby promoting lean manufacturing strategies with limited wastage and significantly reducing the cost burden [58].

### 4.2. Downstream Processes

#### 4.2.1. Contract Manufacturing Organisation (CMO) and Co-Manufacturing Units

CMO and co-manufacturing units can be promoted as a practical option for CM and PBM manufacturers to ensure the quick scaling up of production beyond the pilot scale while cutting infrastructure establishment costs [51,59,60]. A CMO strategy in the CM market concomitantly promotes B2B partnerships, leading to enhanced collaborative efforts at all possible CM value chain entry points for stakeholders’ mutual benefit [51,59,60].

#### 4.2.2. Distribution, International Import and Export, and Licensing

There are promising prospects for export and import trading in the international markets for Indian CM products [59]. Indian CM companies and CMOs who are keen to expand their consumer base abroad may look for suitable local partners to facilitate exports, imports, distribution, licensing, and the propagation of joint ventures [6].

#### 4.2.3. Regulatory and Safety Certifications for CM in India

There is a dire need to establish a regulatory and safety framework for the efficient management of the production of CM ingredients and products in the Indian market. Specified authorities and organisations could monitor cell banking strategies, CM cell-line collection, proliferation, and differentiation protocols by conducting periodic inspections of cell banks and cell culturing facilities to ensure compliance with set regulations [17]. Further, commercialisation strategies could be effectively managed by appropriate quality assurance and CM ingredient and product processing certifications issued by relevant authorities to manage the CM industry (Table 3).

## 5. Product Focus for CM Development

The cultivated meat market in India can be primarily prospected based on a product focus involving: (a) the use of a marketing strategy involving product launching and area- and customer-wise marketing plans; and (b) the promotion of marquee business partnerships [1]. Early-stage Indian startups possess a higher degree of operational flexibility compared with well-established companies and can concentrate on the production of low-cost, serum-free, and antibiotic-free cell culture media ingredients and growth factors. [35,61]. Further, medium-sized startups and established firms might play a prominent role in the fabrication of customised bioreactors, experiment with novel scaffolding techniques, including nanofibers, 3D bioprinting, and extrusion methods, develop species-specific CM cell lines, and explore biobanking strategies for CM cell lines [35,61]. The Indian CM ecosystem possesses skilled professionals whose potential is untapped and academic and industry partners offering a myriad of career opportunities in the upstream and downstream production stages of the CM industry [20].

### 5.1. CM Product Mind Map

**Figure 3:**
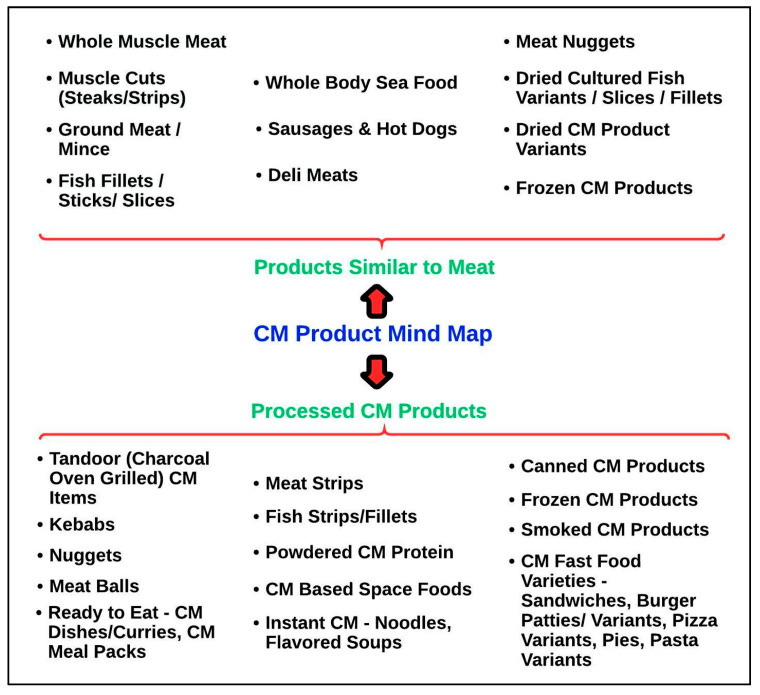
CM Product Mind Map (created with Lucidchart.com).

Figure 3 presents a sustainable CM product mind map that caters specifically to Indian cuisine and the consumer market. CM manufacturers can easily surpass the limitations of the anatomical, physiological, and diversity factors associated with animal-specific products. Hence, innovation can be propagated by formulating novel CM products tailored to Indian cuisine as an alternative to the replicative CM counterparts of age-old meat products [62]. Herein, we categorise different CM products based on mind maps for the innovation and industrialisation of CM in India (Figure 3).

### 5.2. CM Products Similar to Meat

CM product varieties that essentially replicate meat products with a similar appearance, texture, and Indian flavour and aroma have been the prime focus of several recent and ground-breaking developments in the cultivated meat sector [63]. Interestingly, CM product variants can be categorised based on the species of origin, the molecular structure, and the composition, unlike conventional meat products pertaining to animal species alone. The taste component of meat is quite a complex entity involving chemical interactions between different molecules that occur in a highly versatile and unpredictable manner during cooking [62]. To create unique, customised CM ingredients and a product portfolio for a typical Indian consumer, there is an obvious need to delineate the signatures of molecular interactions associated with the texturisation and taste appeal relevant to native cuisine [62].

### 5.3. Processed CM Products

Processed CM products are specialised formulations designed to cater to the needs of the prepared meals market domain comprising heat-and-serve entrees and hotel and fast-food chain environments suitable for Indian cuisine [63]. Innovation pertaining to this segment includes the creation of ingredients and additives for making CM products that can retain their texture, shape, and flavour profile during storage in a refrigerator, thawing, and the heating/cooking of CM-based meals [9]. Promoting processed CM products will support the large-scale production of CM in India, enhancing the price parity for these unique, custom-designed variants in the sector of heat-and-serve entrees and rolling out CM variants in the fast-food sector, capturing economic societies and lower-cost markets [51,63].

### 5.4. Blended Meat Products

Blended CM products offering a healthy nutritional profile with high fibre content and lower-saturated fat, cholesterol, and calories when coupled with essential nutrients can be created to be sold in food supply retail markets [64]. Products can be formulated with (a) CM blended with pre-extruded textured proteins; or (b) CM blended with whole-plant ingredients, including pulses, vegetable extracts, and spices [54,64].

## 6. Consumer Insights and Behaviour towards CM

**Figure 4:**
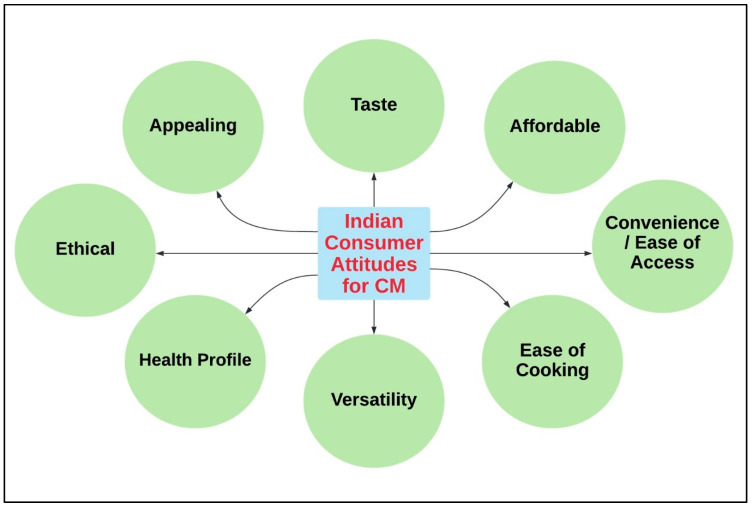
Indian consumer attitudes toward CM (created with Lucidchart.com).

The Indian CM market can identify early adopter segments and categories of enthusiastic innovators willing to include CM products in their diet by carrying out: (a) focus group studies adhering to a specific geographical area or a particular parameter; and (b) a wide range of survey-based analyses [65]. It is a common notion that groups with a high degree of interest in CM are overrepresented by youngsters—Generation Z, millennials, libertarians, flexitarians, and urbanites. India is highly multicultural; hence, prior familiarity with and the opportunity to taste CM products in culturally relevant food dishes are envisioned to enhance consumer adoption rates [65]. Consumers are becoming increasingly aware of the potential advantages of CM in terms of a reduction in environmental impacts, livestock resources and maintenance, and the mitigation of public health risks [65,66]. The most promising consumer attitudes in India towards CM are appeal, taste, affordability, convenience/ease of access, ease of cooking, versatility, health profile, and ethical aspects [67] (Figure 4).

### Concerns about CM

Indian consumers have certainly expressed concerns about taste matching, affordability, safety matters, and the origin of CM [65]. Earlier findings noted that effective science communication strategies, including prior familiarity with, narrative building for, and transparency about CM products, could ease Indian consumers’ concerns significantly. The CM product naming criteria used to appeal to consumer preferences should include criteria that differentiate CM products from conventional meat products [66].

## 7. Conclusions and Future Outlook for the Indian CM Market

Indian companies and skilled workers have tremendous potential to be at the forefront in the global CM market, driving novel gastronomical experiences and cultural appeal with transformative CM products of relevance to food sustainability. Stakeholders, including investors, entrepreneurs, and strategic partners, possess a broad range of opportunities at various stages of the CM development process, leading to a substantial ability to capitalise on the global shift occurring in meat production [36]. Public-private partnerships among universities, companies, and non-profit organisations (e.g., Good Food Institute India, Humane Society International) are accelerating the momentum needed to set up funding initiatives and research infrastructure [19]). Fundraising and proofs-of-concept for products play a pivotal role in accelerating the Indian CM industry [8]. B2B enterprises stationed at India’s strategic locations can promote the co-manufacture and distribution of low-cost and specialised CM components, including cell line development, cell banking, and bioreactor operations [59,60]. Synergism between the CM industry and innovative technologies, including artificial intelligence, bioprinting, data science and analytics, design thinking, the internet of things, lean methodologies and applications, lean manufacturing, machine learning, machine intelligence, and nanotechnology, promises to foster CM innovations in India and globally. Cost-effective and sustainable CM products can be created to address food sustainability and security aspects of the meat industry with the upcoming research initiatives from governmental and private sectors coupled with the enormous funding possibilities [8].

Multinational companies and startups from Asia (Singapore, Korea), Mediterranean (Israel), Pacific (Japan, Australia, New Zealand), South America, North America (United States of America, Canada), European Union and the United Kingdom can entrust on the enormous technical infrastructure, skills and production capabilities of developing Indian CM industry [51]. In December 2020, Singapore Food Agency (SFA) gave the world‘s first regulatory approval for the sale of CM branded as “Good Meat-Cultured chicken bites” manufactured by USA based startup ‘Eat Just Inc’ [68]. The historic tasting event of CM nuggets held in Singapore received positive acclaim [68,69]. These are the most significant milestones and can be noted as an ethical advancement in the global food industry. These landmark developments clearly show that CM based products are not just technologically feasible at the research stage but also feasible as a scaled product for human consumption [68,69]. Indian CM industry can adopt similar regulatory approval frameworks followed by SFA for commercial sale of CM based products in India and abroad [68]. There is tremendous scope for multiple engagements with geographically closer matured alternative protein ecosystems such as Singapore for mutual growth, development and expansions both in the public as well as private space. This can also attract Indian and foreign stakeholders to promote fruitful B2B collaborations, contract manufacturing units, import and export business in India and the global CM industry [59,60,68].

## Figures and Tables

**Table 1:** Value-chain entry points for CM in India.

S. No	Value-Chain Entry Point	Prospective Strategies/Growth Avenues	Stakeholders Involved
**Upstream/Production**			
01	Cell Line Development	Species-specific/native cell line isolation and biobanking Carbon-footprint-free cell line immortalisation and maintenance techniques Automated cell screening systems Genetic engineering for unique cell line variants	DBT India; AIC-CCMB; ICT Mumbai; CECA; NRC Meat; GFI India; HSI India; Clear Meat; IIT Guwahati
02	Cell Culture Media and Ingredients	Animal-free origin ingredients Growth factor mimetics Novel molecule screening platforms Fermentation additive products Micro or nanofluidic cell culture systems	DBT India; AIC-CCMB; ICT Mumbai; CECA; NRC Meat; GFI India; HSI India; Clear Meat; IIT Guwahati; RichCore Life Sciences
03	Scaffolding	Biocompatible hydrogels, nanofibers, nanotubes 3-D bioprinting, extrusion technologies Photopolymerisation Self-directed architecture	DBT India; AIC-CCMB; ICT Mumbai; CECA; NRC Meat; GFI India; HSI India; Clear Meat; IIT Guwahati; MyoWorks
04	Bioreactors/Cell Cultivation Systems	Media recycling and filtration Automated continuous bioreactors Artificial intelligence (AI), machine learning (ML), and internet of things (IoT)-based sensors for the control and monitoring of the growth environment Automation of bioprocessing units	DBT India; AIC-CCMB; ICT Mumbai; CECA; NRC Meat; GFI India; HSI India
**Downstream/Collateral**			
05	Manufacturing/Production	Facility design, construction, management, and maintenance Co-manufacturing/co-packing units Customised Indian CM product variants Product branding and white label process	DBT India; ICT Mumbai; CECA; GFI India; HSI India
06	Sales and Distribution	Indian cultural expertise Branding strategies, marketing, and sales Product consulting and brokerage system Export and import market Global expansion paradigms	DBT India; ICT Mumbai; CECA; GFI India; HSI India
07	Supply Chain Management	Quantity and quality assurance Packaging and distribution channels Effective local and global sourcing	DBT India; ICT Mumbai; CECA; GFI India; HSI India
08	Regulatory and Business	Regulatory and safety certifications Intellectual property and patents Business and legal consulting Entrepreneurship and technology transfer	DBT India; AIC-CCMB; ICT Mumbai; CECA; IIT Guwahati; GFI India; HSI India; Clear Meat; BIV; Gastrotope; String Bio 19

Table 1 Legends: DBT India, Department of Biotechnology, India; AIC-CCMB, Atal Incubation Centre—Centre for Cellular and Molecular Biology; ICT Mumbai, Institute of Chemical Technology (ICT) Mumbai; CECA, Centre of Excellence in Cellular Agriculture; NRC Meat, National Meat Research Centre; GFI India, Good Food Institute India; HSI India, Humane Society International (HSI) India; BIV India, Big Idea Ventures India; IIT Guwahati, Indian Institute of Technology Guwahati; ONGC, Oil and Natural Gas Corporation.

**Table 2:** Stakeholders in India’s CM industry: organisations/companies and their contributions to the CM industry in India.

S. NO	Organisation	Type	Ongoing Activities	Website	References
1	Department of Biotechnology (DBT), India	Govt	Intellectual and funding resources to stakeholders for the acceleration of CM R&D in India.	http://dbtindia.gov.in/ accessed on 15 September 2021	[23]
2	Atal Incubation Centre—Centre for Cellular and Molecular Biology (AIC-CCMB)	Govt and Technology Incubator	Hae been awarded a grant of $640,000 from DBT India for research on in vitro muscle stem cell cultivation. Collaborations: GFI India, DBT India, HSI India, NRC Meat.	http://aic.ccmb.res.in/ accessed on 15 September 2021	[19,23,37,38]
3	Institute of Chemical Technology (ICT) Mumbai.	Govt	Setting up the Centre of Excellence in Cellular Agriculture. Collaborations: GFI India, DBT India.	https://www.ictmumbai.edu.in/ accessed on 15 September 2021	[23]
4	Centre of Excellence in Cellular Agriculture (CECA)	Govt	ICT Mumbai and GFI India set up the CECA in Maharashtra for CM R&D in India. Collaborations: GFI India, ICT Mumbai, DBT India.	N/A	[23]
5	National Meat Research Centre (NRC Meat)	Govt	Research on the culture and differentiation of muscle stem cells in serum-free media. Collaborations: AIC-CCMB, GFI India, HSI India, DBT India.	https://nrcmeat.icar.gov.in/ accessed on 15 September 2021	[19,39]
6	Good Food Institute (GFI) India	Non-Profit Global	They publish detailed CM guides, white papers, and research articles. Provide services to CM industry stakeholders in govt, academic, and corporate sectors. Collaborations: AIC-CCMB, NRC Meat, HSI India, ICT Mumbai. DBT India, BIV, Ashika Group.	https://www.gfi.org.in/ accessed on 15 September 2021	[19,23,38,40]
7	Humane Society International (HSI) India	Non-Profit Global	Funding support for the promotion of open-access publication of in-depth reviews and conceptualisations of accessory technologies for accelerating CM industrialisation in India. Collaborations: AIC-CCMB, GFI India.	https://www.hsi.org/ accessed on 15 September 2021	[19,37,38]
8	Big Idea Ventures	Private Accelerator	Creation of the BIV-Ashika India Alternative Protein Fund and an accelerator to promote CM startups. Collaborations: GFI India, Ashika group, Kolkata.	https://bigideaventures.com/ accessed on 15 September 2021	[40]
9	Gastrotope	Farm-to-fork accelerator	Incubating Clear Meat for CM research. Collaborations: Clear Meat.	https://gastrotope.com/ accessed on 15 September 2021	[41]
10	RichCore LifeSciences	Private	R&D of ‘Non-Animal-Origin’ (NAO) recombinant proteins including media components, reagents, and excipients for the CM industry. Collaborations: DBT India.	http://www.richcoreindia.com/ accessed on 15 September 2021	[21]
11	Clear Meat	Private	R&D and patent filed for the development of technology for cultured meat (chicken mince). Collaborations: DBT India, JNU, New Delhi, Gastrotope.	https://www.clearmeat.com/ accessed on 15 September 2021	[41,42,43]
12	MyoWorks at the Society for Innovation and Entrepreneurship (SINE) incubator at IIT Mumbai.	Private	Awarded a grant of INR 5 million/USD 67,314 from DBT India. R&D on the creation of edible, vegan scaffolds for the CM industry. Collaborations: DBT India, IIT Mumbai.	NA	[44]
13	IIT Guwahati	Govt	R&D and technology patent obtained for a muscle progenitor cell culture on edible scaffold materials. Collaborations: DBT India.	https://www.iitg.ac.in/ accessed on 15 September 2021	[45]
14	String Bio19	Private	Patented a String-Integrated Methane Platform (SIMP) for utilisation of methane as a carbon source for bacterial growth leading to the production of alternative protein to be developed as CM ingredients. Collaborators and Investors: Ankur capital, ONGC, Seventure Partners, Kitven, and Srinivasa Hatcheries.	http://www.stringbio.com/ accessed on 15 September 2021	[46]

**Table 3:** Regulatory and Safety Certifications for the Indian CM Industry.

	Certification/Organisation/Authority	Website
Quality assurance	Hazard Analysis Critical Control Point (HACCP) by the National Centre for HAACP	https://www.haccpindia.org/ accessed on 15 September 2021
	International Organisation for Standardisation (ISO:9000)	https://www.iso.org/home.html accessed on 15 September 2021
	General Society of Surveillance (SGS) India	https://www.sgsgroup.in/ accessed on 15 September 2021
	Good Manufacturing Practices (GMP) India	https://fssai.gov.in/cms/hygiene-requirements.php accessed on 15 September 2021
	Good Hygienic Practices (GHP) India	https://fssai.gov.in/cms/hygiene-requirements.php accessed on 15 September 2021
	Export Inspection Council of India (EIC)	http://eicindia.gov.in/ accessed on 15 September 2021
Product processing	Prevention of Cruelty to Animals Act 1960	http://www.awbi.in/policy_acts_rules.html accessed on 15 September 2021
	Bureau of Indian Standards (BIS) 2007	https://bis.gov.in/ accessed on 15 September 2021
	The Food Safety and Standards Act 2006 (FSS Act)	https://www.fssai.gov.in/cms/food-safety-and-standards-act-2006.php accessed on 15 September 2021
Authorities	Agricultural and Processed Food Products Export Development Authority (APEDA)	https://apeda.gov.in/apedawebsite/ accessed on 15 September 2021
	Food Safety and Standards Authority of India (FSSAI)	https://www.fssai.gov.in/ accessed on 15 September 2021
	Department of Animal Husbandry and Dairying	http://dahd.nic.in/ accessed on 15 September 2021
	Ministry of Agriculture and Farmer’s Welfare	http://dare.nic.in/ accessed on 15 September 2021
	Ministry of Food Processing Industries	https://mofpi.nic.in/ accessed on 15 September 2021
	Marine Products Export Development Authority (MPEDA)	https://mpeda.gov.in/ accessed on 15 September 2021

## Data Availability

Not applicable.
